# How effective is CBCT-guided endodontic access over ‘brain-guided’ accesses, and is this a likely addition to the general dental practitioner’s armamentarium?

**DOI:** 10.1038/s41432-025-01116-9

**Published:** 2025-01-31

**Authors:** Sandeep Pai, David Edwards, Greig Taylor

**Affiliations:** 1 ST4 in Restorative Dentistry, Newcastle-upon-Tyne Dental Hospital, Newcastle-upon-Tyne, UK; 2 FHEA. School of Dental Sciences, Newcastle University, Framlington Place, Newcastle upon Tyne, NE2 4BW UK; 3 PGCert Health Economics, MClin Res, M Paed Dent RCPS(Glasg), PhD, School of Dental Sciences, Newcastle University, Framlington Place, Newcastle upon Tyne, NE2 4BW UK

**Keywords:** Root canal treatment, Dental diseases

## Abstract

**Design:**

Non-randomised prospective single-arm controlled clinical trial. The main inclusion criteria for both guided and freehand access groups was pulp canal obliteration (PCO). All teeth underwent cone-beam CT (CBCT) scan prior to access. Null hypothesis was that there is no difference in technical failure between guided and unguided access. The primary outcome was canal location success as a discrete measure (found, not found, perforated). The secondary outcome was conservativeness of drill pathway using discrete measures (optimal precision, acceptable precision, technical failure (included canal not found and perforation)). Patients underwent one subsequent annual follow-up.

**Case selection:**

Patients attended for consultation at one centre (University Hospital Leuven, Belgium). PCO extent was assigned using periapical radiograph followed by CBCT. Cases graded as ‘high difficulty’ (via qualitative assessment) by briefed endodontists within the centre were selected for the study. Control group was taken from historical records as assigning an active patient to the freehand over the guided group was considered unethical.

**Data analysis:**

Sample size calculation to achieve conventional 80% power and statistical significance of 0.05% was undertaken, mandating 60 teeth per group. An analysis including all data, without matching, was performed by a generalized linear model for binary data using a logit link function with the primary outcome (canal found or not found/perforation) as response variable and the technique (guided or freehanded) as explanatory variable. *p*  ≤  0.05 was considered statistically significant. Blinding of operators was not possible. Teeth were matched (paired) per group to achieve group homogeneity.

**Results:**

Guided access yielded a 98.3% (*n* = 59/60) success rate with only one case unsuccessful (canal not found). In comparison, freehand access yielded 81% (*n* = 59/73) success rate, with 9.5% (*n* = 7/73) of teeth with canals unfound, and 9.5% (*n* = 7/73) of teeth perforated. Null hypothesis rejected given statistical significance of results (*p* = 0.011).

**Conclusions:**

Cases presenting with PCO that undergo guided access show optimal outcomes both from an endodontic and from a conservational perspective, with significant increased chances of canal location in a conservative manner. Freehand correction in guided access cases can be used to inform changes to initial guided access if the canal is not found.

## A Commentary on

**Torres A, Dierickx M, Lerut K** **et al**.

Clinical outcome of guided endodontics versus freehand drilling: A controlled clinical trial, single arm with external control group. *Int Endod J* 2024; **58:** 209–224.

**GRADE Rating:** 

## Commentary

Guided surgery has been utilised in medicine and dentistry with multiple applications^1^. The advent of three-dimensional radiographic scans to assist the clinician in assessing endodontic cases has also been invaluable for practitioners^2^.

Guided endodontic access is currently in its early stages of implementation. Pre-operative CBCT scan and fabrication of a tooth-borne guide with sleeve sits over the pulp chamber roof to support endodontic access^3^. The approach is similar to a dental implant surgical guide when preparing for implant osteotomy. Guided access shows promise, particularly in cases where coronal pulpal anatomy is obscured by anatomical changes.

Sample size was informed by means of appropriate statistical testing, but given the lack of prior studies, arbitrary values to define failure/ success rates were based on clinician opinion. Blinding of operators was not possible given the nature of the intervention, and ethical concerns were raised in assigning patients to an unguided group given the assumed superiority of guided access, therefore use of a bank of previously treated patients prior to the advent of guided access was required as the sole alternative management strategy, albeit being sub-optimal for contemporaneous comparison.

Homogeneity of the samples between groups was noted. Patients underwent assessment and special investigations in a similar manner and were treated by similar grade operators with the use of a standardised equipment armamentarium, which may not be employable by practitioners in other units, suggesting that the results of this workflow are not necessarily externally valid.

Workflow for guided access was specified within the paper. One note of contention is that enamel was removed over the access area prior to the guided access to facilitate use of the carbide bur; it is unclear whether the guided access was offset from this to allow for accurate drilling depth.

A superior canal location rate (98%) was achieved using guided access. No teeth within this group incurred perforation. Only one out of 60 teeth’s canal remained unlocated, but this was treated by means of apical surgery. This was a stark contrast to the unguided control group which exhibited an 81% canal location rate suggesting the superiority of guided access over unguided. No parameters with regards to time taken undertaking the access was noted which could help further prove the efficacy of guided access over conventional freehand access. On the other hand, use of guided access may prove more efficacious in complex anatomy cases only and not for routine cases, given the costs of CBCT, laboratory fees for guide fabrication and other requirements.

Some guided access cases required guide removal, peri-operative periapical film and the subsequent unguided ‘righting’ of the access. Mean deviation of 0.45 mm was noted for certain cases at the apical portion of the access, which suggests that even in cases which did not have first-attempt plumb access, that further investigation if required was only required at the apical portion of the access therefore conserving coronal tooth structure.

Guides however may be subject to cumulative inaccuracies with their fabrication which would be detrimental to the overall conservative nature of guided access. Such issues may be pre-empted by an initial pre-root canal treatment appointment where the passivity and accuracy of the guide may be checked^4^.

The present study has limited external validity, as interventions were undertaken in the same sole unit by a sole operator suggesting there may be an element of bias and lack of applicability of findings to the general population. The study also mandates that specialists must use guided access, which appears paradoxical when considered with the idea behind guided access, namely facilitating access in challenging cases which would be ideal for the general dental practitioner.

Unfortunately, no CBCT was taken following obturation owing to the lack of clinical necessity; this would have been ideal to assess the three-dimensional nature of the access to confirm its conservative nature to further the case for guided access’ superiority in maintaining tooth tissue.

Endodontic microsurgery must not be disregarded as a modality to treat the obliterated canal tooth^5^. Should guided access be unsuccessful, the success rates based on this study could prompt the operator to expedite apical surgery. This would maintain tooth substrate and negate the likelihood of perforation.

Guided access appears to be progressing at an exponential rate. Augmented reality, namely dynamic photogrammetry-guided navigation without the need for a hard guide may be the next stage of endodontic access evolution; however, there is a paucity of clinical or research evidence to support this approach.

In summary, based on this study, guided endodontic access is superior to unguided, free-hand access for cases that would be considered markedly challenging. This could offer, the best possible outcome for an already endodontically-compromised tooth. Given its relative novelty, further studies and investigation is essential to further inform this statement. Guided access offers both clinical and non-clinical (e.g. reduction in time needed for access) benefits, but it must be recognised that endodontic access remains only one of the determinants in ensuring a sound endodontic outcome. Guided access is therefore not a panacea and operator discretion, experience and ingenuity are invaluable.

Practice points

- In cases where root canal anatomy is obscured, CBCT may be used not only to assess the case, but also to produce a milled guide which one may drill through to access the pulp space. This is still in its early stages but shows promise with regards to maintaining tooth tissue and integrity whilst gaining access to diminutive root canal anatomy.
- The field of guided access is advancing, and it is currently recommended that such practice remains within the field of specialist practice.
